# Building consensus on the utility of central and peripheral physiological markers for neuropsychiatric symptoms of dementia: a Delphi Study

**DOI:** 10.1002/alz.091349

**Published:** 2025-01-09

**Authors:** Ka Sing Paris Lai, Samira Choudhury, Sanjeev Kumar, Amer M. Burhan

**Affiliations:** ^1^ Ontario Shores Centre for Mental Health Sciences, Whitby, ON Canada; ^2^ Scarborough Health Network, Scarborough, ON Canada; ^3^ University of Toronto, Toronto, ON Canada; ^4^ Temerty Faculty of Medicine, University of Toronto, Toronto, ON Canada; ^5^ Centre for Addiction and Mental Health, Toronto, ON Canada

## Abstract

**Background:**

Neuropsychiatric symptoms (NPS) of dementia are a heterogenous group of non‐cognitive symptoms and behaviors that occur in up to 90% of individuals with the condition. Characterizing NPS is a major issue and current methods are unreliable as they rely on subjective observations. Automatic identification of behaviors using central and peripheral physiological markers may be helpful to detect behaviors, allow for early intervention, and prevent critical incidents in patients with dementia.

**Methods:**

This study will use a modified Delphi methodology to develop a guide for the use of central and peripheral markers that can be used for the detection, assessment, and monitoring of treatment response for persons with dementia exhibiting NPS in institutions. Experts including clinicians, educators, and researchers will be recruited internationally to participate as expert panellists. This study will review the current literature regarding the use of central and peripheral markers and NPS (e.g. wearable devices, mobile tracking devices, heart rate variability, skin conductance, and electroencephalography). A list of survey items will be developed and presented to the expert panellists for review and feedback. Survey items will also be refined accordingly based on panellist feedback. It is anticipated that there will be a total of 3 rounds of the modified Delphi process.

**Implications:**

NPS can cause distress to patients and caregivers and increase the risk of injuries to the patients themselves and those in their vicinity. It is difficult for caregivers in care facilities or at home to continuously monitor the persons with dementia. If expert recommendations can be achieved about the use of markers for the detection, assessment, and monitoring of treatment response for NPS, clinicians will be able to better provide early intervention and deliver personalized treatment plans for persons with dementia exhibiting NPS.

